# Proteomics analysis of human intestinal organoids during hypoxia and reoxygenation as a model to study ischemia-reperfusion injury

**DOI:** 10.1038/s41419-020-03379-9

**Published:** 2021-01-18

**Authors:** Anna M. Kip, Zita Soons, Ronny Mohren, Annet A. M. Duivenvoorden, Anjali A. J. Röth, Berta Cillero-Pastor, Ulf P. Neumann, Cornelis H. C. Dejong, Ron M. A. Heeren, Steven W. M. Olde Damink, Kaatje Lenaerts

**Affiliations:** 1 Department of Surgery, Maastricht University Medical Centre; NUTRIM School of Nutrition and Translational Research in Metabolism, Maastricht, The Netherlands; 2 Maastricht MultiModal Molecular Imaging Institute (M4I), Maastricht University, Maastricht, The Netherlands; 3 Department of General, Visceral and Transplantation Surgery, RWTH Aachen University Hospital, Aachen, Germany

**Keywords:** Mechanisms of disease, Proteomics

## Abstract

Intestinal ischemia-reperfusion (IR) injury is associated with high mortality rates, which have not improved in the past decades despite advanced insight in its pathophysiology using in vivo animal and human models. The inability to translate previous findings to effective therapies emphasizes the need for a physiologically relevant in vitro model to thoroughly investigate mechanisms of IR-induced epithelial injury and test potential therapies. In this study, we demonstrate the use of human small intestinal organoids to model IR injury by exposing organoids to hypoxia and reoxygenation (HR). A mass-spectrometry-based proteomics approach was applied to characterize organoid differentiation and decipher protein dynamics and molecular mechanisms of IR injury in crypt-like and villus-like human intestinal organoids. We showed successful separation of organoids exhibiting a crypt-like proliferative phenotype, and organoids exhibiting a villus-like phenotype, enriched for enterocytes and goblet cells. Functional enrichment analysis of significantly changing proteins during HR revealed that processes related to mitochondrial metabolism and organization, other metabolic processes, and the immune response were altered in both organoid phenotypes. Changes in protein metabolism, as well as mitophagy pathway and protection against oxidative stress were more pronounced in crypt-like organoids, whereas cellular stress and cell death associated protein changes were more pronounced in villus-like organoids. Profile analysis highlighted several interesting proteins showing a consistent temporal profile during HR in organoids from different origin, such as NDRG1, SDF4 or DMBT1. This study demonstrates that the HR response in human intestinal organoids recapitulates properties of the in vivo IR response. Our findings provide a framework for further investigations to elucidate underlying mechanisms of IR injury in crypt and/or villus separately, and a model to test therapeutics to prevent IR injury.

## Introduction

Intestinal ischemia-reperfusion (IR) is a potentially life-threatening condition associated with a range of clinical conditions including acute mesenteric ischemia, shock and major surgery. Reperfusion of the ischemic intestine paradoxically aggravates tissue injury. Many biological processes are implicated in the complex pathophysiology of IR injury, including the oxidative stress response, cell death programs, epithelial barrier breach, innate and adaptive immune responses, and the interplay with the luminal microenvironment^1,2^. Although previous work using animal IR models^3^ and, in the last decade, human in vivo IR models^4,5^ have provided important insights in understanding pathophysiological processes that occur during IR, the inability to translate findings to effective therapies contributes to continued high mortality rates of intestinal ischemia^6,7^. A physiologically relevant in vitro model is crucial to thoroughly investigate mechanisms of IR-induced epithelial injury and test potential therapies, and, hence, to eventually achieve an improved patient outcome.

IR-induced damage starts at the tip of the villi, and may, as the duration of ischemia increases, continue towards the crypt^8–10^. Reperfusion following prolonged ischemia resulted in apoptosis of Paneth cells in a human IR model^11^. Mature enterocytes at the villus tips are most susceptible to IR, which has been classically explained by the oxygen gradient along the crypt-villus axis with decreasing oxygen tension towards the villus tip^12^. In addition, mature enterocytes are physiologically in a pro-apoptotic state which makes them prone to be shed from the villus^13^. This mechanism is crucial for homeostasis, but may also contribute to increased susceptibility for IR-induced cell death compared to immature epithelial cells. These differences in IR response between crypt and villus, emphasizes the importance of a model that enables the study of both compartments.

Conventional two-dimensional cell lines do not reflect the complex intestinal architecture and composition and are far from translatable to the human setting. The recently established intestinal organoid model has attracted attention as an in vitro tool to study intestinal (patho)physiology. Intestinal organoids are three-dimensional epithelial structures that recapitulate the cellular diversity and many functions of the intestinal epithelium^14–16^, which makes them closer to normal human physiology and thus superior to immortalized cell lines. Furthermore, organoids resemble the genetic signature of original tissues^15,17–19^, and have been shown to exhibit a personalized proteome profile^20^. Next to the purpose of studying epithelial biology (e.g., tissue renewal and niche function), organoids have been used for disease modeling, e.g., cancer, hereditary diseases and infectious diseases^21–24^, and as tools for personalized cancer therapy^25^ and regenerative medicine^26^.

In this study, we used human small intestinal organoids to model IR injury by exposing them to hypoxia–reoxygenation. As induction of a differentiated organoid phenotype allows for the separation of crypt and villus regions^22,23^, the differences in molecular response to IR in the distinct regions could be investigated. We aimed to characterize the different organoid phenotypes, as well as decipher protein dynamics and molecular mechanisms of IR injury in crypt- and villus-like human intestinal organoids, by using a system-wide mass spectrometry (MS)-based proteomics approach.

## Results

### Differentiation of human small intestinal organoids into crypt-like and villus-like organoids

**Fig. 1:**
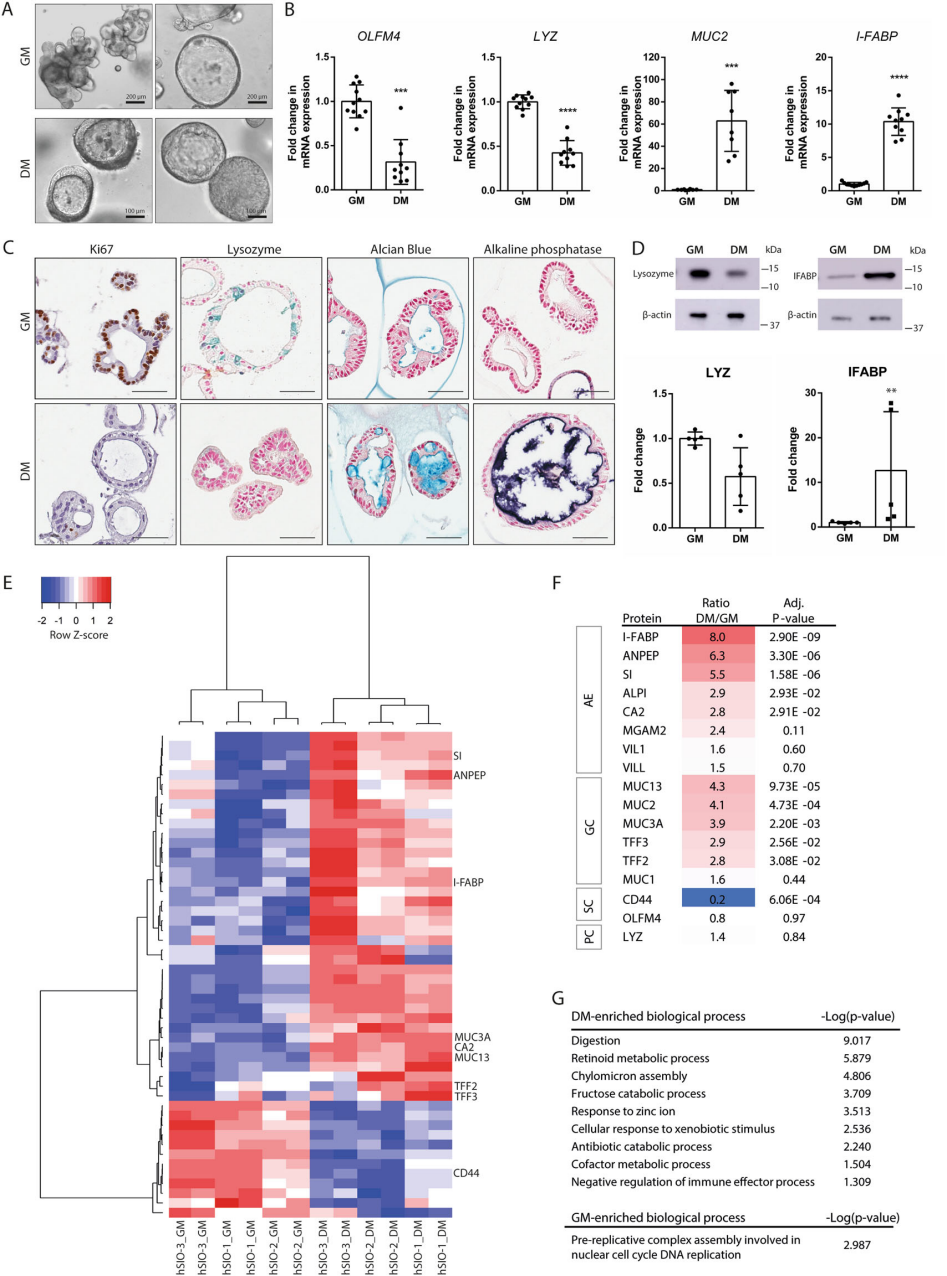
Characterization of crypt-like and villus-like human small intestinal organoids. **A** Brightfield images representing different phenotypes of hSIOs cultured in complete growth medium (GM) for 12 days (upper panel, scale bar = 200 µm) and differentiated hSIOs grown in GM for 7 days followed by 5 days in differentiation medium (DM) (lower panel, scale bar = 100 µm). **B** mRNA expression of crypt cell markers OLFM4 (stem cells) and LYZ (Paneth cells) and villus cell markers I-FABP (absorptive enterocytes) and MUC2 (Goblet cells). Data were normalized to B2MG and ACTB reference genes, and reported as relative expression as compared to GM (set at 1). Results were obtained from 4 different hSIO lines (*n* = 10–11, Mean ± SD, ***P < 0.001, ****P < 0.0001, Mann Whitney U-test). *OLFM4* Olfactomedin, *LYZ* Lysozyme, *I-FABP* Intestinal fatty acid-binding protein, *MUC2* Mucin 2. **C** Representative stainings for intestinal protein markers in sections of GM- and DM-cultured hSIO. Proliferation marker Ki67, lysozyme-containing Paneth cells, Alcian Blue-stained Goblet cells, and Alkaline phosphatase staining of the brushborder. Scale bar = 50 µm. **D** Western blot analysis of lysozyme and IFABP. Band intensity was quantified and normalized using β-actin as loading control. Relative expression is shown as mean ± SD (*n* = 5, obtained from 3 different hSIO lines; **P < 0.01, Mann Whitney U-test). Full blots can be found in Fig. S1. **E** Heatmap showing relative change of differentially expressed proteins (*P* < 0.05). Proteins with on/off expression are excluded from the heatmap and can be found in Table S1. *n* = 6 from 3 different hSIO lines. **F** List of known protein markers for intestinal cell-types, with respective abundance ratio (DM/GM) and adjusted *p*-value. AE absorptive enterocytes, GC Goblet cells, SC stem cells, PC Paneth cells. **G** Functional enrichment analysis showing GO biological processes significantly enriched (adjusted *p*-value < 0.05, corresponding to −log(*p-*value) > 1.3) in DM organoids and GM organoids. Protein lists for enrichment analysis: upregulated in DM *p*-value < 0.05; upregulated in GM *p*-value < 0.1. See also Table S1 for the lists of proteins used for enrichment analysis.

Culture of human small intestinal organoids (hSIOs) in growth medium (GM) for 12 days resulted in either a multilobular or cystic organoid phenotype (Fig. 1A, upper panel). To induce differentiation, organoids were cultured in differentiation medium (DM) for 5 days following 7 days GM, which resulted in a cystic phenotype either with or without a clear lumen (Fig. 1A, lower panel). The differentiated state was demonstrated by higher gene expression of *I-FABP* and *MUC2* compared to undifferentiated hSIOs (Fig. 1B). Alcian blue staining of mucus-containing goblet cells, a more intense staining of brush border enzyme alkaline phosphatase (Fig. 1C) and higher protein expression of I-FABP (Fig. 1D, Fig. S1) confirmed the presence of goblet cells and enterocytes following differentiation. Interestingly, 20–40% of DM-cultured organoids showed villus-like structures pointing towards the lumen (Fig. 1C, alkaline phosphatase staining). Downregulation of stem cell marker *OLFM4* (Fig. 1B) and a lower number of KI67-expressing cells (Fig. 1C) was indicative for a reduced proliferative potential in DM-cultured organoids. Paneth cell marker *LYZ* was significantly higher (Fig. 1B) and lysozyme staining appeared more pronounced in undifferentiated organoids (Fig. 1C), however, lysozyme protein levels were not significantly different (Fig. 1D, Fig. S1).

To further characterize undifferentiated (GM-cultured) and differentiated (DM-cultured) organoids, we implemented a quantitative proteomics approach. A total of 2182 unique proteins were identified across three different hSIO lines, of which 109 proteins were differentially expressed (75 upregulated, 32 downregulated) in DM compared to GM organoids (Table S1). Clustering analysis showed a clear separation between DM and GM with great similarity between different hSIO lines (Fig. 1E). A list of known cell-type protein markers with their abundance ratios are shown in Fig. 1F. Enterocyte and goblet cell markers were markedly increased in DM-cultured organoids (Fig. 1F), whereas markers for enteroendocrine cells (CMGA, CHGB, SYP) and tuft cells (DLCK1) were not identified. Intestinal stem cell marker CD44 was reduced in DM-cultured organoids. Paneth cell marker LYZ showed no overall significant change. Next, functional differences between GM and DM-cultured organoids were evaluated by GO enrichment analysis. Enriched biological processes in differentiated organoids were predominantly related to digestion and metabolic processes (Fig. 1G). Upregulated proteins in GM (*p*-value < 0.1) were enriched for processes related to DNA replication (Fig. 1G).

Overall, these data indicate that DM induced a villus-like (VL) organoid phenotype enriched for enterocytes and goblet cells, whereas GM-cultured organoids present a crypt-like (CL) phenotype enriched for proliferating cells. This allows for the separate investigation of the crypt- and villus response to hypoxia and reoxygenation.

### Proteomic profiling of the response to hypoxia–reoxygenation in crypt and villus-like organoids

**Fig. 2:**
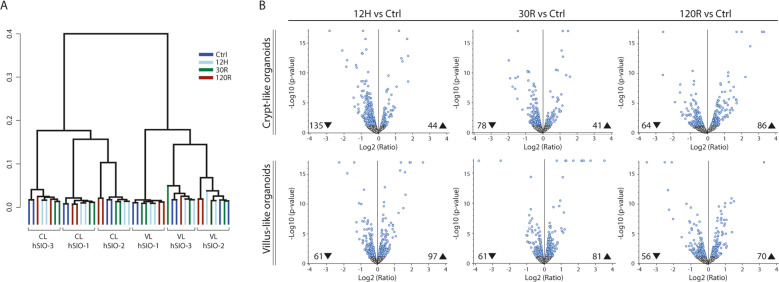
Proteomics analysis of the response to hypoxia–reoxygenation. **A** Hierarchical clustering of the complete proteomics dataset. 12H 12 h of hypoxia, 30 R 30 min of reoxygenation, 120 R 120 min of reoxygenation. **B** Volcano plots for experimental conditions 12H, 30R, and 120R compared to Ctrl in crypt-like and villus-like hSIOs, in which abundance ratio (Log2) is plotted against the *p*-value (−log10). The number upregulated (▲) and downregulated (▼) proteins are indicated in every plot (adjusted *p*-value < 0.05). On/off proteins are not shown and can be found in Table S2. *N* = 6 from 3 hSIO lines.

We next performed in-depth proteomics analysis of the response to hypoxia and reoxygenation (HR), mimicking ischemia and reperfusion, in both CL and VL organoids. The following experimental conditions were investigated: 12 h of hypoxia (12H), 30 and 120 min of reoxygenation (30R, 120R), and no HR (Ctrl). Hierarchical clustering analysis of the complete proteomics dataset revealed that the differentiation state of the organoids (CL versus VL) was responsible for the two main clusters (Fig. 2A). Higher similarity was observed between samples derived from the same hSIO line than between samples in the same experimental HR condition. Biological replicates from all hSIO lines were combined to investigate robust HR-induced proteomic changes. Volcano plots in Fig. 2B show the number of up-and downregulated proteins at 12H, 30R, and 120R compared to Ctrl in CL and VL organoids (See Table S2 for lists of differentially expressed proteins).

### Enrichment analysis of altered biological processes during hypoxia–reoxygenation

**Fig. 3:**
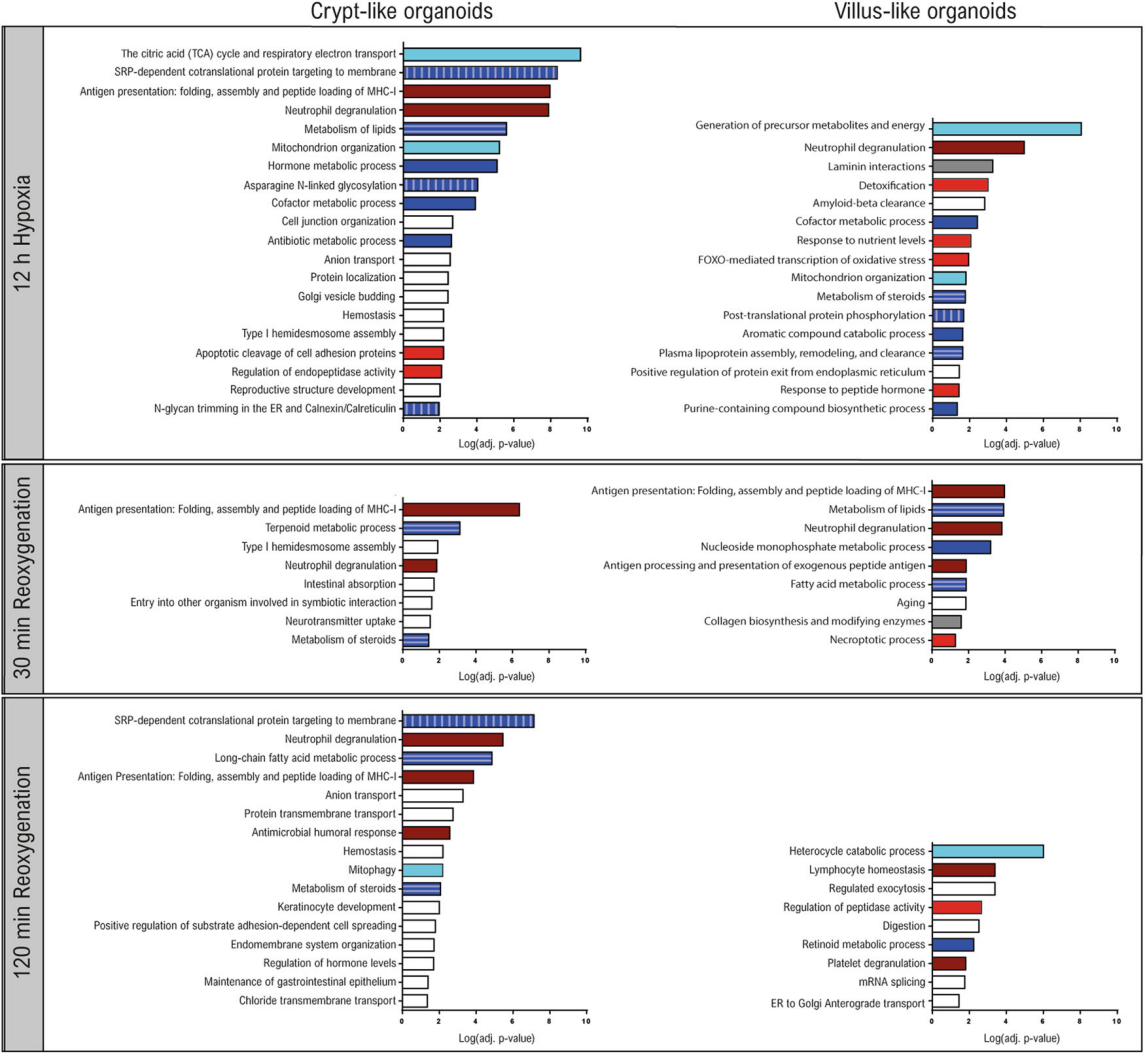
Functional enrichment analysis. Enriched biological processes at 12H, 30R, and 120R compared to Ctrl in CL organoids (left) and VL organoids (right) are shown. A combined list of significantly up- and downregulated proteins was used for analysis, and an adjusted *p*-value < 0.05 was considered statistically significant. Enrichment analysis for GO biological processes and Reactome pathways was performed using METASCAPE. Bar colors indicate groups of selected processes. Processes related to mitochondrial structure and metabolism (light blue), enriched in both CL and VL organoids. Metabolic processes (dark blue): protein metabolism, enriched in CL organoids (vertical stripe); lipid metabolism, in CL and VL organoids (horizontal stripe). Processes related to stress response and apoptosis (red), more pronounced in VL organoids. Extracellular matrix interactions (gray), enriched in VL organoids. Immune response (dark red), enriched in both CL and VL organoids.

Functional enrichment analysis for the combined up- and downregulated proteins was performed to analyze biological processes that are changed in response to HR, and to determine the crypt- and villus response. Enriched processes at 12H, 30R, and 120R compared to Ctrl are shown in Fig. 3 and abundance ratios of selected proteins can be found in Fig. S2.

#### Mitochondrial metabolism and organization

The most significantly enriched biological process at 12H in both CL and VL organoids was related to energy production in the mitochondria. Additionally, the process ‘Mitochondrion organization’ was enriched at 12H in both CL and VL organoids (Fig. 3, light blue bars). Several subunits of the mitochondrial respiratory chain complexes were significantly changing, primarily at 12H. In VL organoids, we observed an upregulation of complex I subunits (NDUFB10 (Fig. S2A), NDUFS5, NDUFV1), and subunits of complex IV (COX17) and V (ATP5PB) (Fig. S2A). A downregulation of complex V subunits (ATP5PB (Fig. S2A), ATP5ME, ATP5PD, ATP5MG) was found in CL organoids at 12H.

With regard to mitochondrial structure, abundances of mitochondrial outer membrane proteins VDAC1 (Fig. S2B), TOMM70 and TOMM22 (Fig. S2B) were decreased in CL organoids.

Interestingly, at 120 R the mitophagy pathway was significantly enriched in CL organoids (Fig. 3). An increased protein expression of autophagy receptor SQSTM1 (Fig. S2B) and decreased abundances of mitochondrial proteins TOMM22, VDAC1 (Fig. S2B) and SLC25A6, suggests occurrence of mitophagy following HR in CL organoids. No changes in mitophagy-related proteins were observed in VL organoids.

#### Protein metabolism

Enrichment analysis revealed regulation of various metabolic processes in both CL and VL organoids (Fig. 3, dark blue). One of the top enriched processes in CL organoids (‘SRP-dependent cotranslational protein targeting to membrane’) is related to protein translation (Fig. 3, vertical striped). Multiple 60 S ribosomal proteins showed increased abundances in CL organoids following HR (e.g., RPL4, RPL6, RPL7a, RPL13, RPL35, RPL18) whereas in VL organoids we observed reduced abundances (e.g., RPL13, RPL4, Fig. S2C) or no difference (e.g., RPL6, RPL18, Fig. S2C). Furthermore, HR decreased expression of translation initiation factor EIF4G1 (Fig. S2C), proteins implicated in protein transport into the ER (SSR3, Fig. S2C) and co-translational *N*-linked glycosylation (RPN1, RPN2 (Fig. S2C), DDOST), as well as proteins involved in quality control of protein folding (CANX, Fig. S2C) and transport of folded proteins (LMAN1).

#### Lipid metabolism

Processes associated with lipid metabolism were enriched at all time points in CL and VL organoids (Fig. 3, horizontal striped). Several proteins involved in fatty acid β-oxidation showed a decreased expression, and included CPT1A (CL; Fig. S2D) and long-chain-fatty-acid-CoA ligases ACSL3, ACSL5 (CL), and ACSL4 (CL, VL; Fig. S2D). In addition, we observed an upregulation of monoglyceride lipase (MGLL; CL), and fatty acid binding protein (FABP2; CL, VL). Changes in fatty acid synthesis included a reduced expression of fatty acid desaturase (FADS2, Fig. S2D) (CL, VL), and enzymes playing a key role in fatty acid elongation (ELOVL1 (Fig. S2D), HSD17B12, TECR) in CL organoids.

#### Cellular stress response

The processes ‘Response to hypoxia’ and ‘Response to oxidative stress’ were not significantly enriched, however, increased transcript levels of well-known HIF1A target VEGF confirmed hypoxic signaling (Fig. S3). In addition, a closer inspection of protein abundances supports regulation of stress signaling. Hypoxic stress-responsive protein NDRG1 (Fig. S2E) was significantly increased at all time points in both CL and VL organoids. Oxidative stress-related proteins (CHCHD2 (Fig. S2E), ERO1A) were increased at 120 R in VL organoids. In CL organoids, proteins involved in protection against oxidative stress (TXNRD2 (Fig. S2E), PPIF) were increased at 120R, whereas in VL organoids a decrease in antioxidant protein SOD2 (12H, 30R) was observed. In addition, several processes associated with response to a stimulus (e.g., ‘Detoxification’, ‘Response to nutrient levels’) and cell death (e.g., ‘FOXO-mediated transcription of oxidative stress’, ‘Necroptotic process’) were enriched in VL organoids (Fig. 3, red bars). The number of cell death promoting proteins showing increased abundances was highest at 120R, and included among others DIABLO (Fig. S2E), LCN2 (VL, CL), BCAP31 (Fig. S2E) and ERO1A (VL). However, contradictory, the well-known apoptosis regulator BAX was decreased at 120R (VL).

#### Extracellular matrix

Processes related to the extracellular matrix (ECM) were exclusively enriched in VL organoids (Fig. 3, gray bars), with increased expression of basement membrane components (COL4A1, COL4A2; Fig. S2F), LAMB1, NID1, NID2 (Fig. S2F)), and enzymes that play a role in collagen crosslinking (PLOD1, PLOD2; Fig. S2F). In contrast, a decreased expression of COL17A1 was observed (Fig. S2F).

#### Immune response

Finally, processes related to both innate and adaptive immune responses, including ‘Neutrophil degranulation’ and ‘Antigen presentation’ were enriched following HR in both CL and VL organoids (Fig. 3, dark red bars).

### Temporal protein profiles

**Fig. 4:**
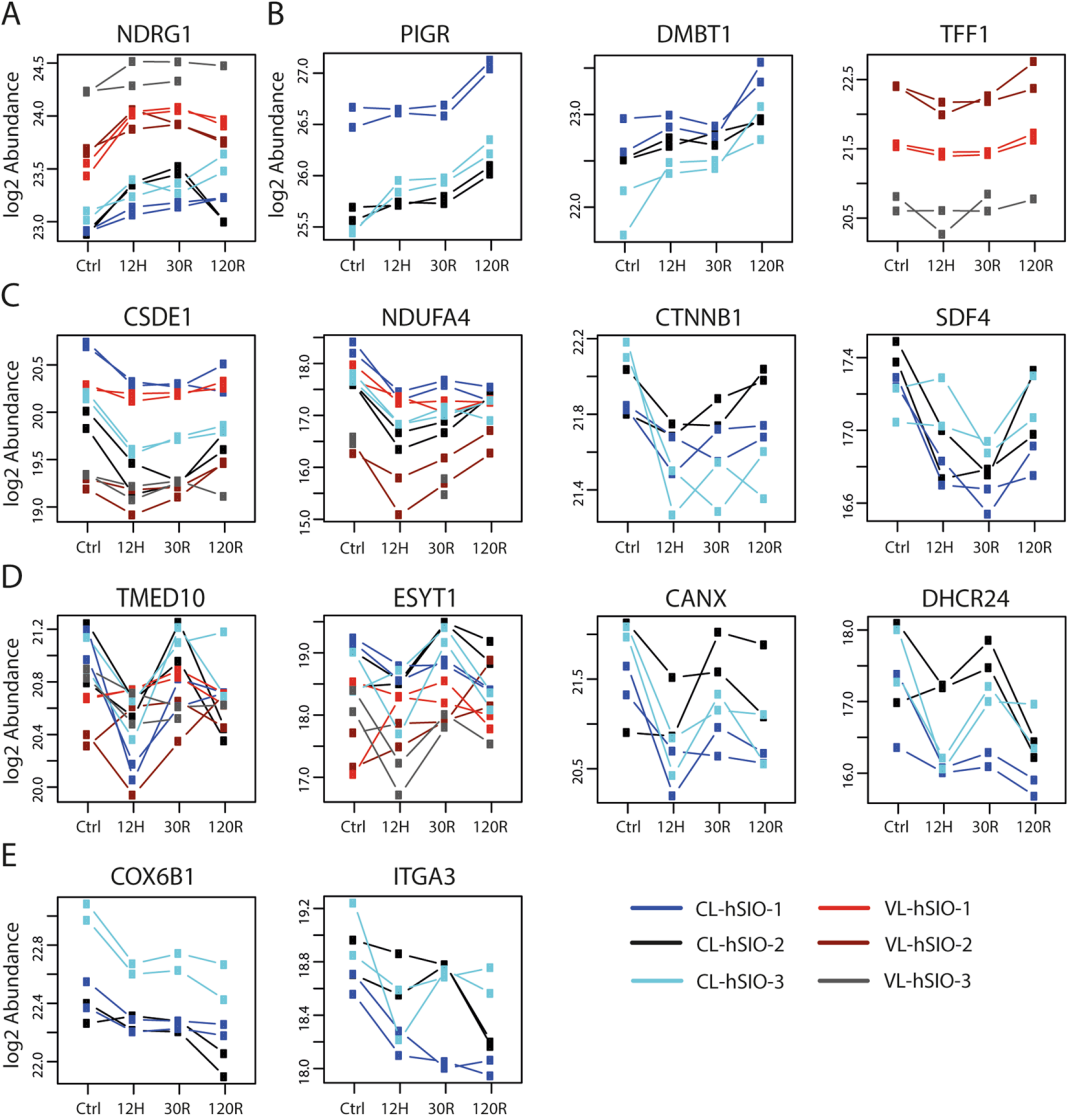
Temporal expression profiles. Proteins showing a consistent temporal profile in both CL and VL organoids, or either CL or VL organoids are shown. Profiles are clustered in five groups which are presented in panels **A**, **B**, **C**, **D**, and **E**. Temporal profile analysis was performed using R package MaSigPro, and temporal profiles were considered statistically different using an *R*-squared ≥ 0.5 and a adjusted *p*-value < 0.3. See also Fig. S4, in which all significant profiles can be found. NDRG1 N-Myc downstream regulated 1, PIGR polymeric immunoglobulin receptor, DMBT1 deleted in malignant brain tumors 1, TFF1 trefoil factor 1, CSDE1 cold shock domain containing E1, NDUFA4 cytochrome c oxidase subunit, CTNNB1 Β-catenin, SDF4 45 kDa calcium-binding protein, TMED10 transmembrane emp24 domain-containing protein 10, ESYT1 extended synaptotagmin-1, CANX calnexin, DHCR24 delta(24)-sterol reductase, COX6B1 cytochrome c oxidase subunit 6B1, ITGA3 integrin alpha-3.

We performed temporal profile analysis to examine which proteins exhibit consistent changes over the course of HR (Fig. S4). We selected biologically interesting proteins showing consistent temporal profiles in all hSIO lines of CL and/or VL organoids by visual inspection (Fig. 4). NDRG1 increased directly following hypoxia in CL and VL organoids with higher baseline expression in VL (Fig. 4A). A profile characterized by increasing expression at 120 R, was observed for PIGR and DMBT1 (CL; Fig. 4B) and TFF1 and TFF2 (VL) (Fig. 4B). Β-catenin (CTNNB1), downstream effector of Wnt signaling, decreased following hypoxia and increased during reoxygenation in CL organoids. A similar profile was observed for SDF4 (CL), CSDE1 (CL, VL), and NDUF4a (CL, VL; Fig. 4C). Different proteins involved in lipid metabolism (DHCR24 (Fig. 4D), ACSL5, NSDHL) exhibit a down-up-down-profile in CL organoids. In addition, this profile was observed for vesicular trafficking protein TMED10, and proteins associated with ER structure and function, namely ESYT1 (CL, VL), CANX (CL; Fig. 4D), and RPN1 (CL). A profile presenting a gradual decrease during HR was observed for cytochrome c oxidases (COX6B1, COX5a; Fig. 4E), and integrin subunits ITGA6 and ITGA3 (Fig. 4E) (CL).

## Discussion

Continued high mortality rates of intestinal ischemia emphasize the need to elucidate molecular mechanisms underlying IR injury. In this study, we demonstrate the use of the hSIO culture system to model IR injury. Proteomics analysis of CL and VL organoids separately has given us novel insights in the response to HR and potential differences between these distinct domains of the intestinal epithelium.

Successful separation of CL and VL organoids was shown by clustering and enrichment analysis of proteome data, as well as analysis of known cell-type specific markers. Consistent with previous hSIO studies^22,23^, hSIOs cultured in GM exhibit a crypt-like proliferative phenotype, whereas DM induced a villus-like phenotype enriched for differentiated enterocytes and goblet cells. Even though we expected a lobular appearance of DM-treated organoids, cystic structures have been observed before in differentiated organoids from human origin^22,23^. Paneth cells, found in both organoid phenotypes, were likely present at the time differentiation was induced, and DM did not further stimulate their differentiation.

The observed clustering per hSIO line rather than experimental HR condition implies that each hSIO line may hold a distinct proteome profile. This assumption is supported by a previous study in which patient-centric clustering of proteome profiles from healthy and tumor colon organoids was reported^20^. In addition, patient-derived tumor organoids resemble the original tumor and recapitulate diversity among patients^17^. The fact that hSIO lines are derived from genetically diverse individuals limits experimental reproducibility in comparison to traditional cell lines, but it is also considered a strength as this reflects interindividual differences.

Adaptation of the cellular metabolic program is required during HR, as is reflected in our functional analysis. Importantly, being major oxygen consumers for energy production, mitochondria and their metabolism are affected by hypoxia. Oxidative phosphorylation is known to adapt by repressing activity of the TCA cycle, and by remodeling protein composition of the electron transport chain^27^. Indeed, different subunits of the respiratory chain were modified after hypoxia. In CL organoids, mainly subunits of complex IV and V were downregulated, which suggests reduced ATP production. The increase in complex I subunits in VL organoids may point to higher ROS-producing capacity^28,29^. These differences between CL and VL organoid responses can be explained by the differences in energy metabolism between epithelial cell types. Proliferating cells rely mostly on aerobic glycolysis which is essential for efficient biosynthesis of macromolecular compounds. In contrast, differentiated cells primarily rely on oxidative phosphorylation^30–32^. In line, intestinal stem cell niche and differentiated cells have been shown to adapt their metabolism by different means in response to metabolic stress^32^.

Next to adaptations in energy metabolism, we report a HR-induced decrease in structural mitochondrial proteins and enrichment of the mitophagy pathway in CL organoids. Mitophagy is a quality control mechanism that selectively removes damaged mitochondria by autophagy^33^, and has been reported to have a protective role against renal and cardiac IR injury^34,35^.

Protein synthesis and ribosome biosynthesis are energy-consuming processes that are suppressed during hypoxia to conserve cellular energy. Changed protein metabolism was observed exclusively in CL organoids, which can be explained by the fact that, in general, dividing cells have more ribosomes and a higher translational activity than non-dividing cells^36^. The increased expression of 60 S ribosomal subunits following HR in combination with no changes in 40 S subunits may be the result of an unbalanced production of 40 S and 60 S ribosomal subunits, as has been described in yeasts in response to stress^37^. The decreased expression of proteins playing a role in *N*-glycosylation suggests that this process is impaired, which may lead to defects in protein folding and degradation^38^. Unfolded protein stress, and subsequent activation of the unfolded protein response, plays an important role in hypoxic and IR injury^5,39–41^. In addition, our data suggests that protein transport into the ER and ER-to-Golgi are affected by HR. Our observations regarding changes in translation machinery, protein folding, and trafficking in CL organoids imply suppression of protein synthesis which is a well-known adaptation mechanism of cells to cope with hypoxia and reoxygenation stress^42^.

Many enzymes involved in lipid metabolic processes were changed during HR and changes were more pronounced in CL organoids. Fatty acids are normally used for oxidation and energy production as well as for synthesis of phospholipids and triacylglycerols^43^. The reduced expression of enzymes involved in fatty acid β-oxidation indicates impaired fatty acid catabolism, which may result in accumulation of free fatty acids and subsequent lipotoxicity and cell death^44^.

Decreased abundances of enzymes catalyzing essential steps in fatty acid elongation and saturation likely affects synthesis and composition of (very) long-chain fatty acids, which are found as components of membrane lipids (glycerophospholipids and spingolipids)^45^. Changes in composition of membrane lipids as a result of altered fatty acid elongation metabolism, has been shown to change membrane properties and increase susceptibility to apoptosis^46^.

Our data were indicative of higher ROS-producing capacity and cell death in VL organoids. Strikingly, proteins involved in protection against oxidative stress and IR injury^47,48^ were exclusively increased in CL organoids. Together, this may indicate that CL organoids possess better protective mechanisms against HR-induced oxidative stress and cell death compared to VL organoids. This reflects the in vivo response in which villi have been shown to be affected first during IR, whereas crypts remain intact for a longer period of time^8^.

Interestingly, in VL organoids, hypoxia induced the expression of various ECM proteins. Even though fibroblasts are the main source of extracellular matrix proteins, intestinal epithelial cells can synthesize ECM proteins as well^49^. Hypoxia-induced collagen synthesis and ECM remodeling by fibroblasts has been reported^50–52^. However, to our knowledge, the effect of hypoxia on ECM remodeling by epithelial cells has not been investigated. Additionally, in CL organoids a gradually decreasing temporal profile was observed for integrins ITGA6 and ITGA3, which suggests that cell–matrix interactions are also affected in CL organoids. A decreased integrin expression may result in reduced adhesion and ECM changes.

Despite the lack of immune cells in our culture system, functional enrichment analysis revealed regulation of immune response related processes. This indicates that HR-induced stress responses interact with immune signaling, even in the absence of immune cells and microbes, which is in agreement with earlier reports^53,54^. Co-cultures of organoids with stromal cells or immune cells could be useful when aiming on specifically investigating the inflammatory response in IR or interactions between host and micro-organism. In this context, as well as for assessment of barrier function, self-organized epithelial monolayers may be a good alternative for 3D culture^55^.

Temporal protein profile analysis highlighted consistent dynamic changes during HR. NDRG1 could be of particular interest in the context of IR injury as it has been shown to suppress pro-survival autophagy pathway and promote apoptosis via modulating ER stress responses^56,57^, which plays an important role in IR injury. Several proteins with an upregulated profile during reoxygenation play a role in mucosal barrier integrity and defense, such as PIGR and DMBT1^58^ in CL organoids, and trefoil factors in VL organoids. Temporal profiles for CTNNB1 and SDF4^59^ reflect halted proliferation in CL organoids during hypoxia, which is resumed during reoxygenation. SDF4 has been shown to play a role in ER-stress induced apoptosis^60^. CSDE1 has not been specifically related with IR injury, but may be of interest considering its role in translational reprogramming^61^. Proteins involved in lipid metabolism (ASCL5, DHCR23, NSDHL) and protein metabolism and transport in the ER (TMED10, ESYT1, CANX, RPN1) showed a down-up-down profile during HR. This may be explained by hypoxic- and oxidative stress-induced metabolic changes at 12H and 120 R, respectively.

In summary, we show that HR-induced protein changes in the hSIO model are involved in biological processes known to be regulated in response to IR. In addition, differences between crypt- and villus responses were highlighted. Most remarkably, cellular stress and cell death associated processes were more pronounced in VL organoids, whereas CL organoids are presumed to possess better protective mechanisms based on upregulation of proteins involved in protection against oxidative stress, and enrichment of the mitophagy pathway. In addition, protein metabolism was only enriched in CL organoids, and HR-induced changes in ECM interaction were most prominent in VL organoids.

We established a model to study the epithelial response to hypoxia and reoxygenation in cultures enriched for crypt and villus cells separately. Our findings demonstrate that hSIO recapitulate in vivo properties of the response to IR and provide a framework for future investigations to decipher underlying mechanisms and test therapeutic targets to prevent or treat IR injury and promote regeneration. Of interest are the protective mitophagy pathway in CL organoids, or specific targets such as NDRG1, SDF4, or DMBT1 as highlighted by temporal profile analysis.

## Methods

### Human intestinal organoid culture

#### Human tissues and ethics

Tissue specimens of healthy small intestine were obtained from patients during pancreaticoduodenectomy at Maastricht University Medical Centre+ or RWTH Aachen University Hospital. The ethics committee of both institutes approved this study (METC 16-4-185, EK 206/09) and written informed consent was obtained. Small intestinal organoid lines derived from 4 patients (average age 72 years; 3 F/1 M), of which 3 for proteomics analysis, were used for this study.

#### Organoid culture and differentiation

Crypts were isolated as described previously by Sato et al.^15^, and embedded in basement membrane extract (BME, Geltrex LDEV-free reduced growth factor basement membrane matrix; Gibco, Carlsbad, CA). Organoids were maintained using growth medium (GM) which contained Advanced Dulbecco’s Modified Eagle’s medium F12 (Gibco) supplemented with Pen/Strep (50 units/ml penicillin and 50 µg/ml streptomycin) (Gibco), 10 mM HEPES (Gibco) and 1x Glutamax (Gibco), with 1x N2 (Gibco), 1x B27 (Gibco), and 50% v/v Wnt3a conditioned medium, 20% v/v Rspondin-1-conditioned medium, 10% v/v Noggin-conditioned medium, 10 mM Nicotinamide (Sigma-Aldrich, St. Louis, MO), 50 ng/ml murine EGF (Gibco), 1.25 mM *N*-acetyl cystein (Sigma-Aldrich), 10 mM Gastrin I (Sigma-Aldrich), 500 nM (TGFβ inhibitor) A83-01 (Sigma-Aldrich), and 10 µM (p38 MAPK inhibitor) SB202190 (Sigma-Aldrich). ROCK inhibitor Y-27632 10 µM (Abmole Bioscience, Houston, TX) was added to the medium only when organoids were generated, after passaging and thawing. Medium was changed every 2–3 days, and organoids were passaged every 10 days using TrypLE^TM^ Express Enzyme (Gibco) followed by mechanical disruption using a glass Pasteur pipette with a narrowed tip. Organoids were frozen 3–4 days after passaging using Recovery^TM^ Cell Culture freezing medium (Gibco), and stored in liquid nitrogen. Cultures were regularly tested for mycoplasma contamination. Organoids used for experiments were passaged at least once after thawing. To induce differentiation, organoids were cultured in GM for 7 days followed by differentiation medium (VL) for 5 days. DM contained the same components as GM without addition of Wnt3a-conditioned medium, Nicotinamide and SB202190 as well as a 50% reduction of Rspondin- and Noggin-conditioned medium.

### Exposure to hypoxia–reoxygenation

To mimic human intestinal ischemia-reperfusion injury, crypt-like (12 days GM-cultured) and villus-like (7 days GM- and 5 days DM-cultured) organoids were exposed to hypoxia (<1.0% O_2_, 5% CO_2_) using a O_2_/CO_2_ incubator (Panasonic), followed by reintroduction to 21% O_2_, 5% CO_2_ (reoxygenation phase). At the start of reoxygenation, medium was changed. Different hypoxic periods, ranging from 2 to 24 h, were examined as it has been previously reported that it may take 4–24 h to reach adequately reduced O_2_ levels in the medium after placing cells in hypoxic conditions^62,63^. The optimal duration of hypoxia was determined based on the stress response (HIF1A target VEGFA and unfolded protein stress signaling) and morphological changes (unpublished data). For the current study, organoids were harvested after 12 h hypoxia (12H), after 30 and 120 min of reoxygenation (30 R and 120 R), and without hypoxic exposure (Ctrl).

### Quantitative real-time PCR

Total RNA was isolated using TRI Reagent (Sigma-Aldrich) according to the manufacturer’s instructions. RNA concentrations were measured with a DeNovix DS-11 spectrophotometer and 750 ng RNA was used for reverse transcription into cDNA using the SensiFast cDNA Synthesis kit (Bioline GmbH, Germany). Quantitative real-time PCR analysis was performed on the LightCycler480 (Roche) using a three-step program (40 cycles). SensiMix SYBR Hi-Rox kit (Bioline GmbH) was used for amplification. Data were processed using LinRegPCR software (version 2016.1). The geometric mean of reference genes beta-2-microglobulin (*B2MG*) and beta-actin (*ACTB*) was used for normalization. Primer sequences are listed in Table S3.

### Immunohistochemistry

Organoids were collected in Cell Recovery Solution (Corning, New York, USA) to remove the BME. Then they were fixed in Unifix 4% paraformaldehyde (Klinipath, Duiven, The Netherlands) for 30 min and washed with PBS. Subsequently, organoids were first embedded in HistoGel (Thermo Scientific, Waltham, MA), followed by dehydration, paraffin embedding, and sectioning (5 µm thickness, Leica microtome).

Paraffin-embedded organoid sections were deparaffinized, and endogenous peroxidase activity was blocked using 0.6% hydrogen peroxide in methanol for 15 min. Antigen retrieval was performed in 10 mM citrate buffer (pH 6.0) at 90 °C for 20 min. Non-specific antibody binding was blocked with 5% BSA in PBS, and sections were incubated with the primary antibodies for 1 h at room temperature. Next, sections were incubated with biotin-conjugated secondary antibodies for 30 min, followed by incubation with avidin–streptavidin complex (Vectorlabs, Burlingame, CA). Antibody binding was visualized with 3,3′-diaminobenzidine (DAB; Dako, Glostrup, Denmark*)* or HistoGreen (Linaris-Biologische Producte, Werheim-Bettingen, Germany). Sections were counterstained with hematoxylin and mounted using Entellan (Merck Millipore, Burlington, Massachusetts, USA). The following primary antibodies were used: lysozyme (Rabbit, 1:5000, Dako), Ki67 (Mouse, 1:200; Dako). Secondary antibodies rabbit anti-mouse (Dako) and swine anti-rabbit (Dako), both biotin-labeled, were used at a 1:500 dilution.

In addition, acidic mucins in goblet cells were stained with Alcian blue solution for 30 min at room temperature. To stain alkaline phosphatase, a brush border enzyme, sections were incubated with a mixture of 4-Nitro blue tetrazolium chloride, 4-toluidine salt, and alkaline phosphate buffer in a humid chamber at 37 °C for 30 min. Both histochemical stainings were counter-stained with Nuclear-fast red. All stainings were digitalized using Aperio CS2 scanner (Leica Microsystems)using a 20x magnification.

### Western blot

Organoids were harvested in Cell Recovery Solution (Corning), followed by multiple ice-cold PBS washes to remove the BME. RIPA lysis buffer supplemented with protease inhibitors (Roche, Mannheim, Germany) and PhosSTOP (Roche), was added to the organoid pellet. Whole-cell lysates were incubated on ice for 20 min and vortexed every 5 min for 60 s. Then, the lysate was centrifuged at 16,000 *g* for 15 min at 4 °C, and the supernatant was collected. Total protein concentrations were determined using the Pierce BCA Assay kit (Thermo Fisher, Rockford, IL).

Laemmli buffer was added to the lysate, and samples were heated for 5 min at 95 °C. Equal amounts of protein were loaded on a 4–20% mini-PROTEAN TGX Stain-Free Gel (Bio-Rad, Hercules, CA) and separated by electrophoresis. Proteins were transferred to a Transblot Turbo PVDF membrane (Bio-Rad) using the Trans-Blot Turbo Blotting System (Bio-Rad). After blocking with 5% non-fat dry milk, membranes were incubated overnight at 4 °C with primary antibodies against: IFABP (rabbit, 5 µg/ml, in-house antibody), Lysozyme (rabbit, 1:2000, Dako) and β-actin (mouse, 1:10000, Sigma-Aldrich). After washing with TBS-Tween 20 (0.1%), membranes were incubated for 1 h at room temperature with HRP-conjugated anti-mouse (1:15000; Jackson ImmunoResearch, West Grove, PA) or anti-rabbit (1:15000, Jackson ImmunoResearch) secondary antibodies. Signals were developed using SuperSignal West Pico chemiluminescent substrate (Thermo Fisher) or SuperSignal West Femto Maximum Sensitivity Substrate (Thermo Fisher), and a molecular imager (Amersham Imager 600, GE HealthcareLifeSciences) was used to obtain images. Band intensity was quantified with ImageQuant TL software (version 8.1, GE Healthcare Life Sciences), and normalized to β-actin control.

### Proteomics sample preparation

Organoids were harvested using Cell Recovery Solution (Corning) and multiple ice-cold PBS washes to remove the BME. For protein extraction, a buffer containing 5 M urea (GE Healthcare, Chicago, IL), 50 mM ammonium bicarbonate (ABC) (Sigma-Aldrich) was added to the organoid pellet. Cell lysis was performed by three freeze–thaw cycles, using a −80 °C freezer for freezing and sonication (40 s) in an ultrasonic bath for thawing. Protein concentrations were assessed using Bradford assay, and equal amounts of protein were used for analysis. Protein lysates were reduced with 20 mM Dithiothreitol (DTT) (Sigma-Aldrich) for 45 min, followed by alkylation with 40 mM Iodoacetamide (Sigma-Aldrich) for 45 min in the dark. The alkylation was terminated by 20 mM DDT to consume any excess Iodoacetamide. In solution digestion was performed with a Trypsin/LysC mixture (Promega, Madison, WI), added at a ratio of 1:25 (enzyme:protein), for 2 h at 37 °C in a Thermo Shaker (Grant Instruments, Shepreth, UK) at 250 rpm. The lysate was then diluted to 1 M Urea using 50 mM ABC and further digested at 37 °C at 250 rpm overnight. Addition of formic acid (Biosolve, Valkenswaard, The Netherlands) to a total of 1% terminated the digestion.

### Liquid chromatography mass spectrometry proteomics analysis

Peptide separation was performed on a Thermo Scientific (Dionex) Ultimate 3000 Rapid Separation ultrahigh-performance liquid-chromatography (UHPLC) system equipped with a PepSep C18 analytical column (15 cm, ID 75 µm, 1.9 µm Reprosil, 120 Å). Prior to UHPLC separation, tryptic peptides were desalted on an online installed C18 trapping column. Peptides were separated on the analytical column using a 90 min linear gradient of acetonitrile (5–35%) with 0.1% FA at a 300 nL/min flow rate. Mass spectra were collected on a Q-Exactive HF mass spectrometer (Thermo Scientific) using a data-dependent acquisition method. Raw mass spectra were processed in Proteome Discoverer software (PD, version 2.2, Thermo Scientific). Protein identification was performed using the SEQUEST search engine in combination with the SwissProt Human database (SwissProt TaxID = 9606, v2017-10-25). For further analysis, results were filtered for proteins identified with high confidence (FDR < 1%).

### Statistical analysis

Data were obtained from three to four different organoid lines. Statistical analysis for gene expression (4 hSIO lines) and Western blot data (3 hSIO lines) was performed using Graphpad Prism (version 6.01). A Mann Whitney U-test was used to compare differences between groups (DM vs GM). A *p*-value < 0.05 was considered statistically significant.

Proteome Discoverer software was used to assess the significance of differential protein expression. One-way ANOVA test was performed on abundance ratios between experimental conditions (6 biological replicates in 3 hSIO lines), and *p*-values were corrected using Benjamini–Hochberg (BH) method. A BH-adjusted *p*-value < 0.05 was considered statistically significant. Proteins originating from Geltrex were excluded from further analysis. Functional enrichment analysis of GO biological processes and Reactome pathways was performed using METASCAPE with default settings^64^.

Hierarchical clustering and temporal profile analysis were performed using R version 3.5.1. The hierarchical clustering used Ward’s method and Pearson’s correlation as the distance measure. The heatmap was created using the R package gplots (heatmap.2). For temporal profile analysis, proteins which were differentially expressed in at least one condition (*P* < 0.05) were selected. In addition, proteins were excluded in case there were ≥1 missing values (ND values). For each significant protein, a regression fit was computed using the R package MaSigPro^65^. Linear, quadratic, third, and fourth order profiles were used. Temporal profiles were considered statistically different from a 0-profile using an *R*-squared ≥ 0.5 and a BH-adjusted *p*-value < 0.3.

## Supplementary information

Supplementary Figure Legends

Figure S1

Figure S2

Figure S3

Figure S4

Table S1

Table S2

Table S3

## Data Availability

The mass spectrometry proteomics datasets generated and analyzed during the current study have been deposited to the ProteomeXchange Consortium via the PRIDE partner repository and are available with dataset identifier PXD022999.
