# Supplementary material for: Proteomics analysis of human intestinal organoids during hypoxia and reoxygenation as a model to study ischemia-reperfusion injury
Source: Cell Death Dis. 2021 Jan 18;12(1):95. doi: 10.1038/s41419-020-03379-9 (PMC7813872; doi:10.1038/s41419-020-03379-9)
Supplement: Supplementary file 1 — Supplementary Figure Legends [file 41419_2020_3379_MOESM1_ESM.docx]

**Supplementary Figure Legends**

**Figure S1.** Western blot data for A) IFABP (13-14 kDa) and B) lysozyme (14-15 kDa). β-actin was used as loading control. Precision Plus Protein Standards (Kaleidoscope), with a range 10-250 kDa was used.

**Figure S2.** Log2 abundance ratios for CL (blue) and VL (red) organoids. Selected proteins associated with A) mitochondrial respiratory chain, B) mitophagy, C) protein metabolism, D) lipid metabolism, E) stress response, apoptosis and anti-oxidant defense and F) extracellular matrix are shown.

**Figure S3.** mRNA expression of HIF1A target VEGF in CL organoids (A) and VL organoids (B). Data were normalized to B2MG and ACTN reference genes and reported as relative expression as compared to Ctrl. Results were obtained from 3 hSIO lines (Mean ± SD) **** P<0.0001 ** P<0.01

**Figure S4.** Temporal protein profiles**.** A) significant profiles in at least 3 out of 6 hSIO lines in CL and VL organoids, B) significant profiles in at least 2 out of 3 hSIO lines in CL organoids and, C) significant profiles in at least 2 out of 3 hSIO lines in VL organoids. Proteins which were differentially expressed in at least one condition compared to control (P<0.05) were included in profile analysis. Temporal profiles were considered statistically different from a 0-profile using an R-squared ≥ 0.5 and a BH-adjusted p-value < 0.3. Plots show Log2 abundances during HR. Dark blue; CL-hSIO-1, black: CL-hSIO-2, light blue: CL-hSIO-3, red: VL-hSIO-1, dark red: VL-hSIO-2, grey: VL-hSIO-3.
