# Supplementary figures and images for: Proteomics analysis of human intestinal organoids during hypoxia and reoxygenation as a model to study ischemia-reperfusion injury
Source: Cell Death Dis. 2021 Jan 18;12(1):95. doi: 10.1038/s41419-020-03379-9 (PMC7813872; doi:10.1038/s41419-020-03379-9)

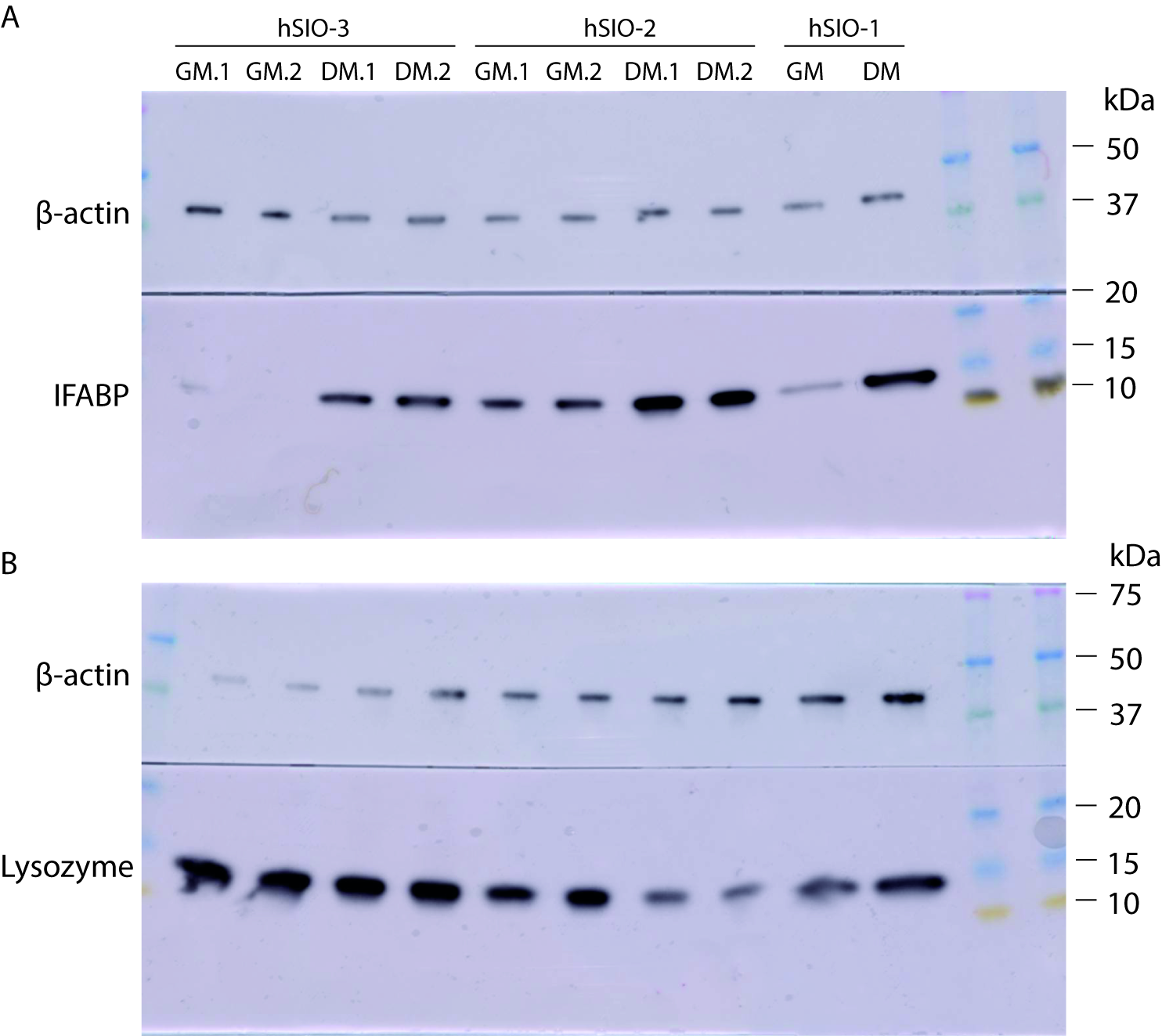

Supplement: Supplementary file 2 — Figure S1 [file 41419_2020_3379_MOESM2_ESM.tif]

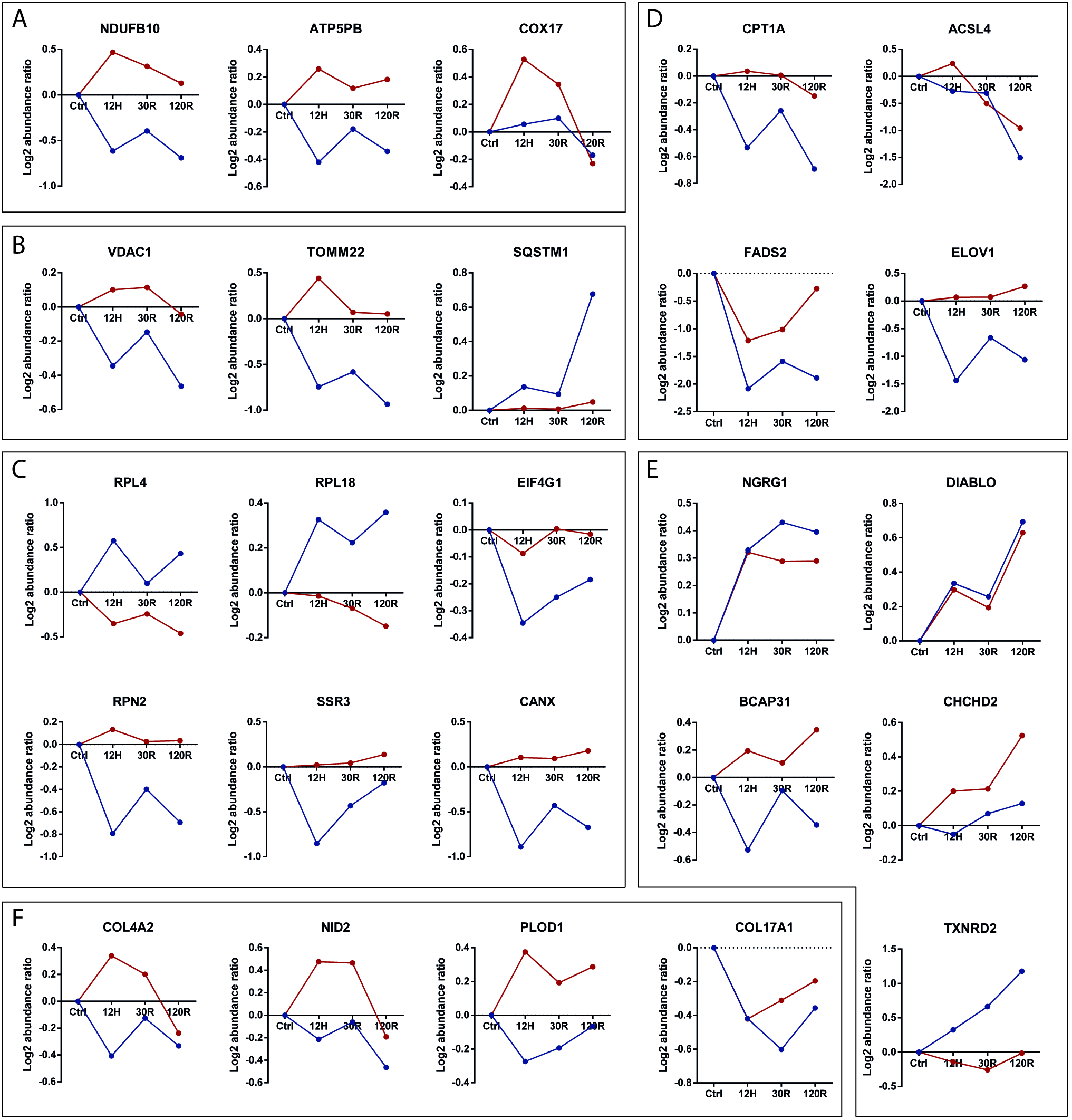

Supplement: Supplementary file 3 — Figure S2 [file 41419_2020_3379_MOESM3_ESM.tif]

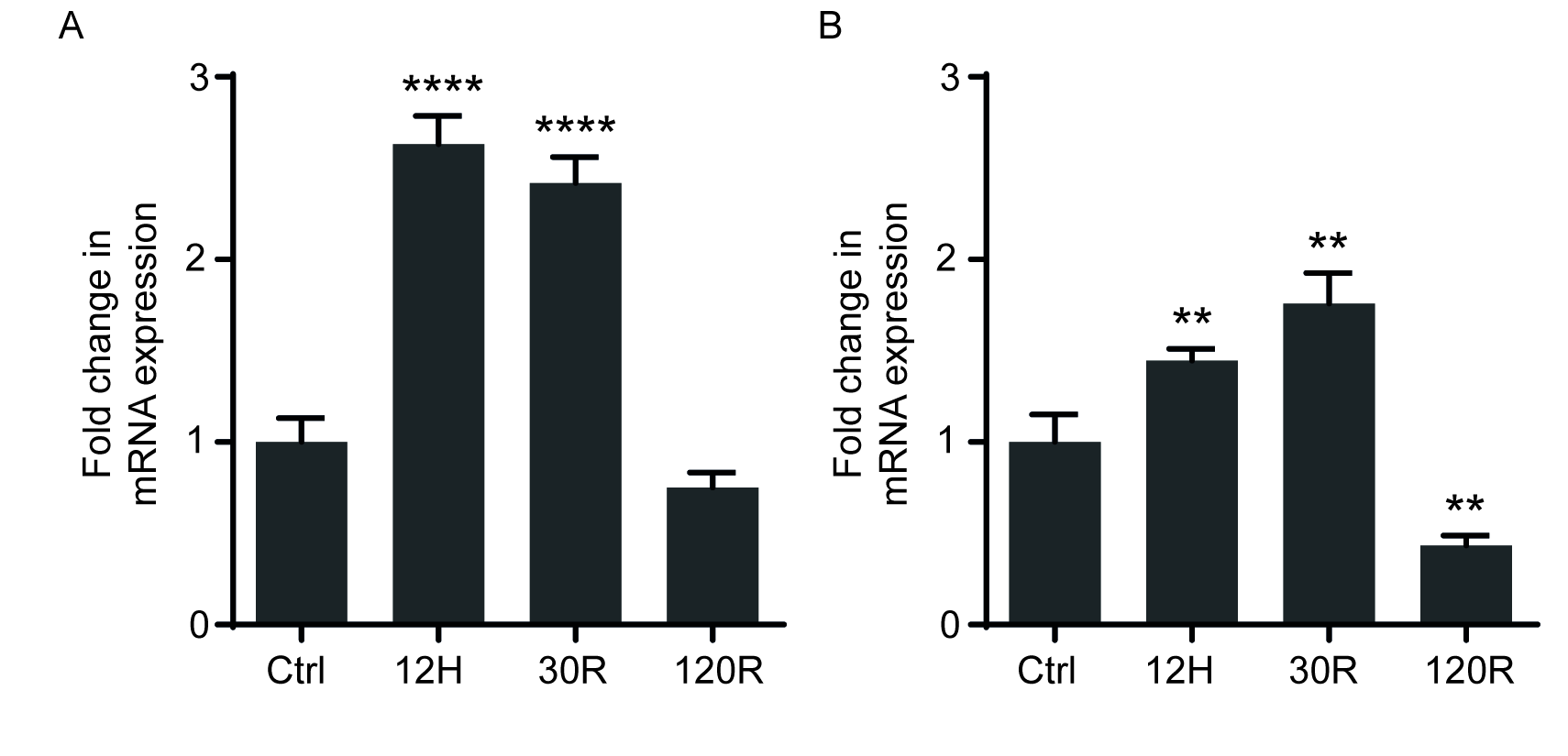

Supplement: Supplementary file 4 — Figure S3 [file 41419_2020_3379_MOESM4_ESM.tif]

**A**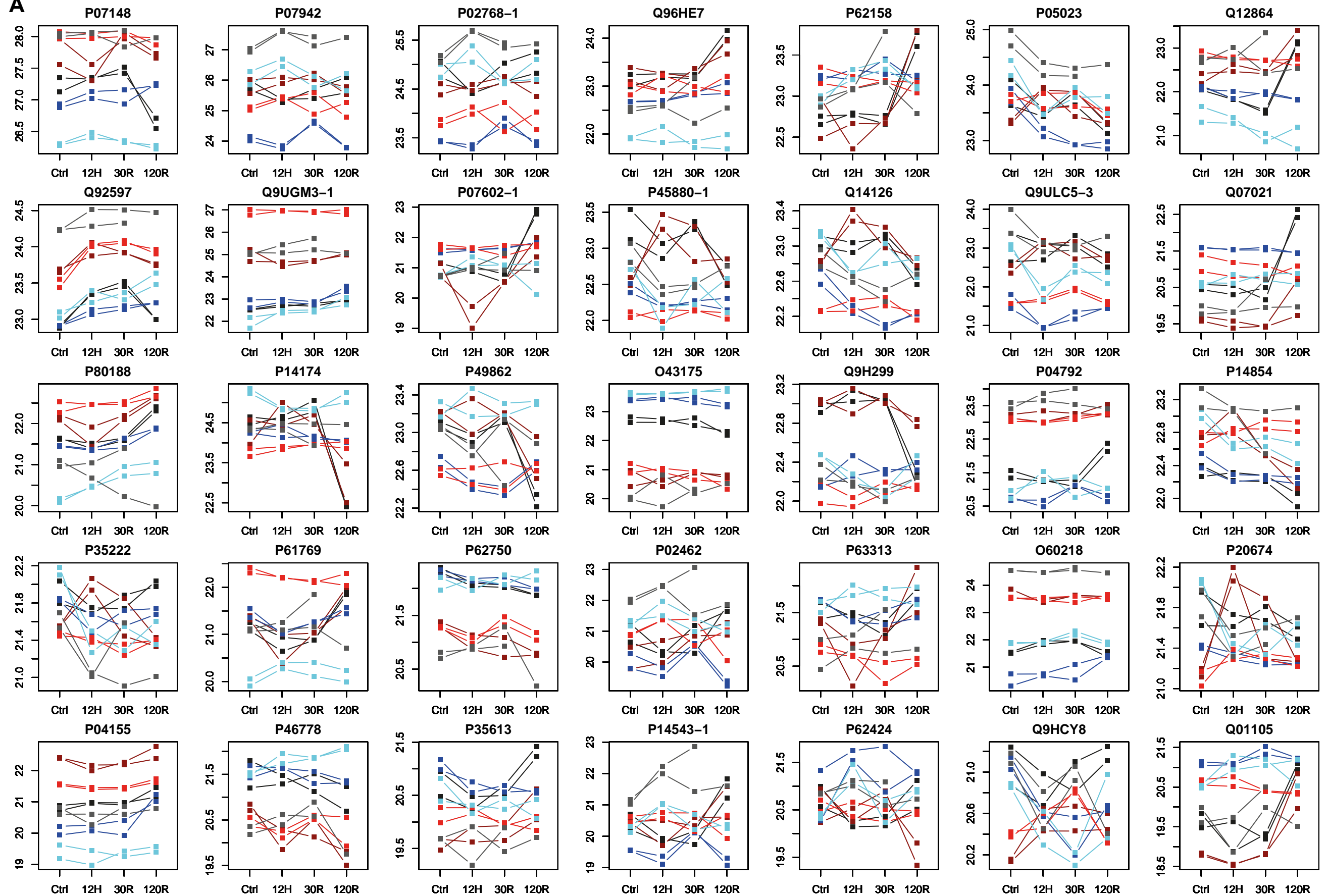

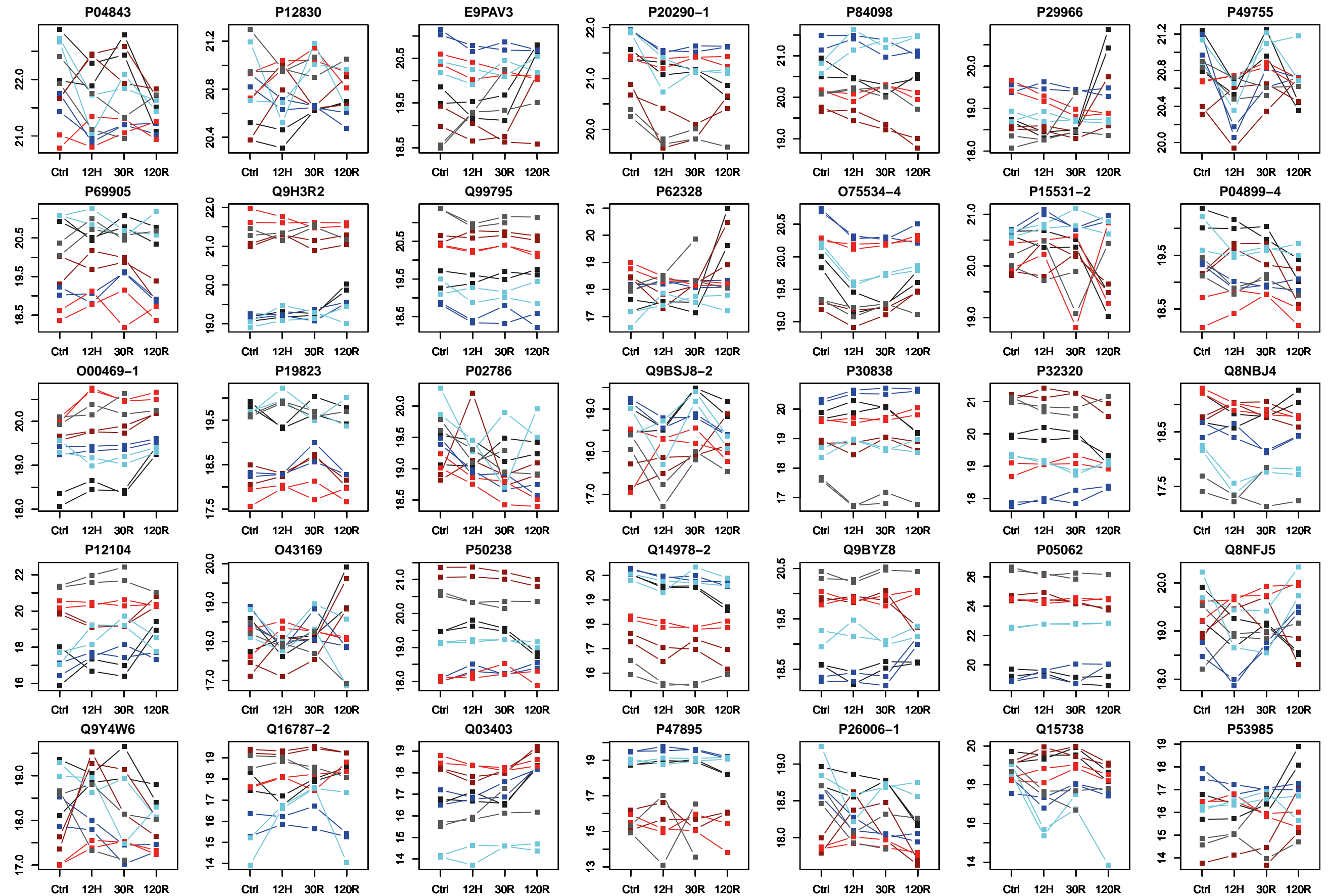

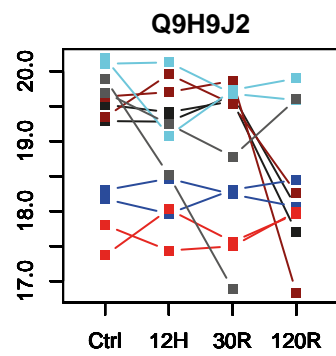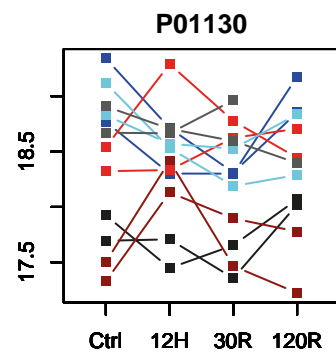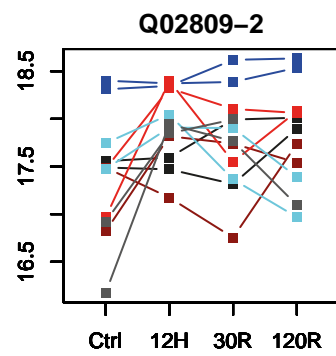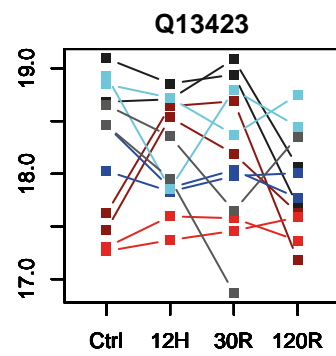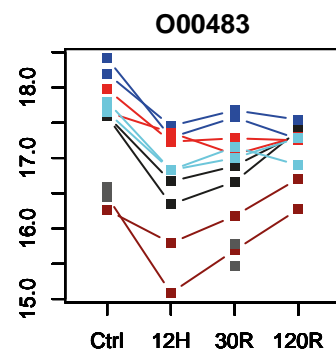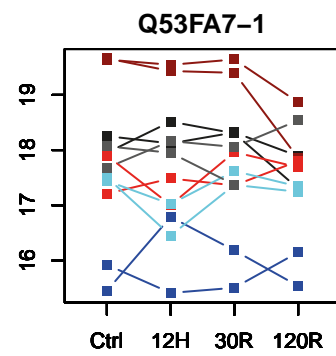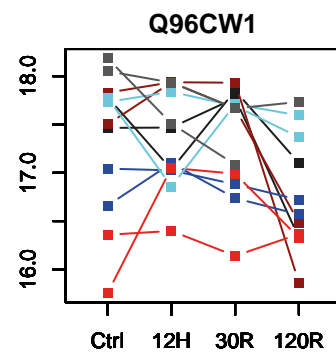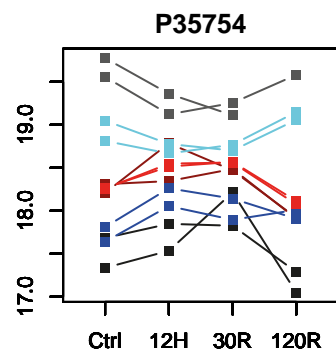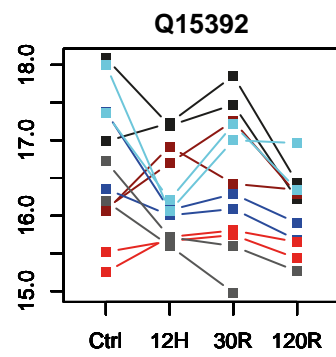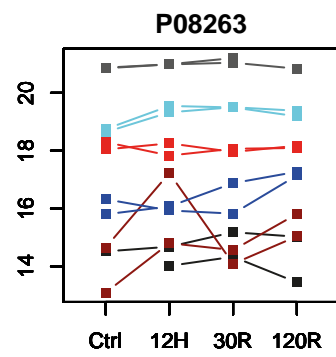

**B**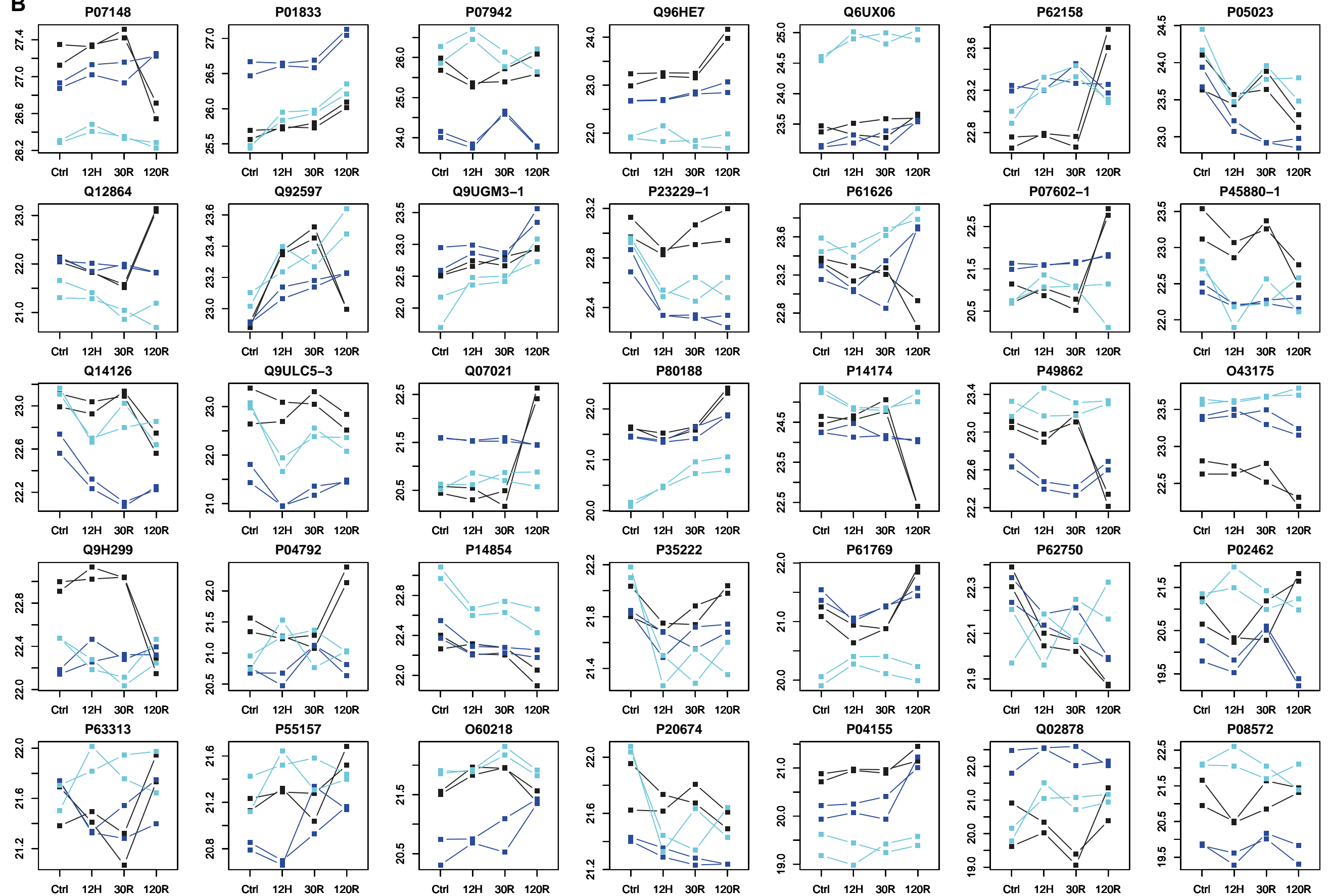

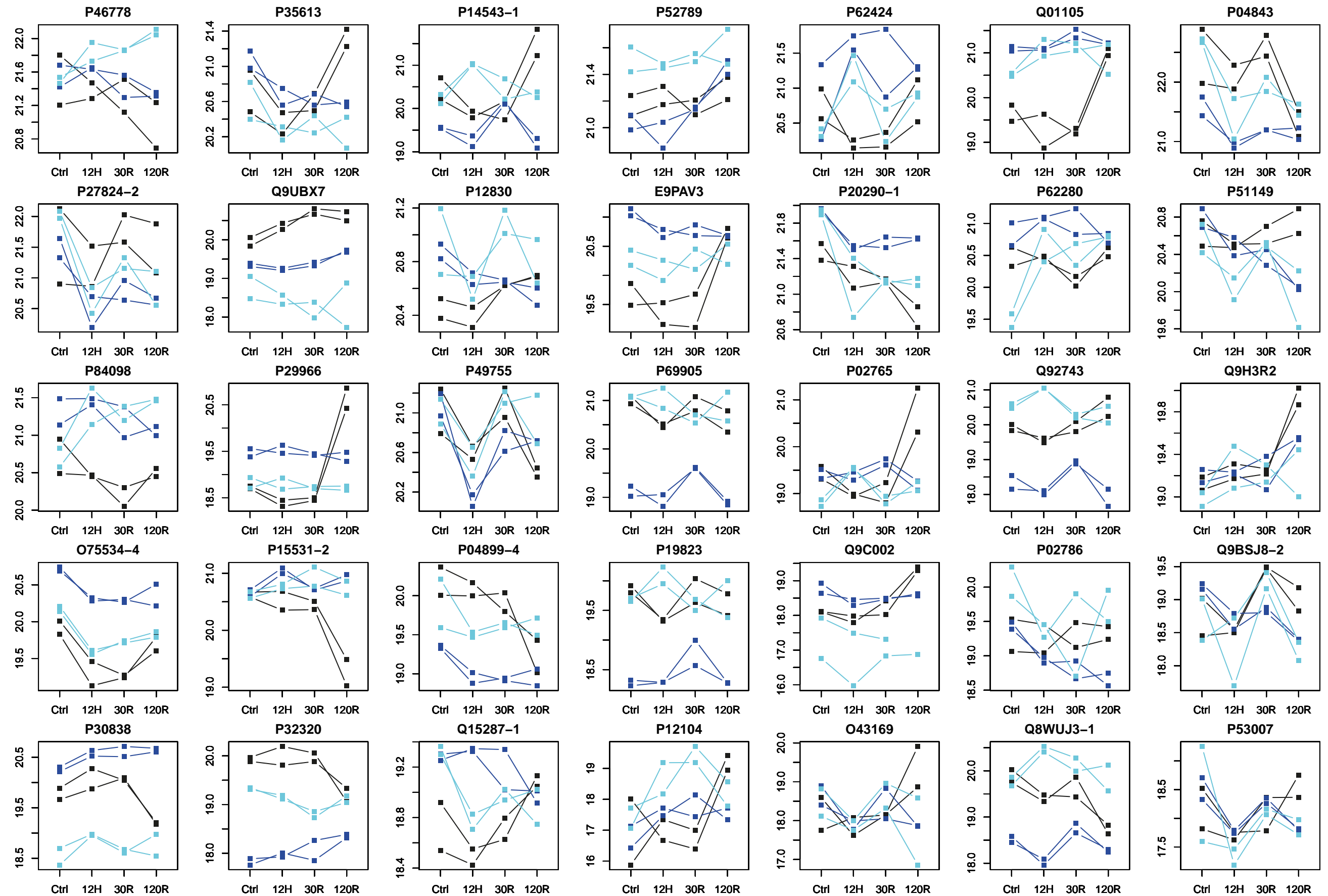

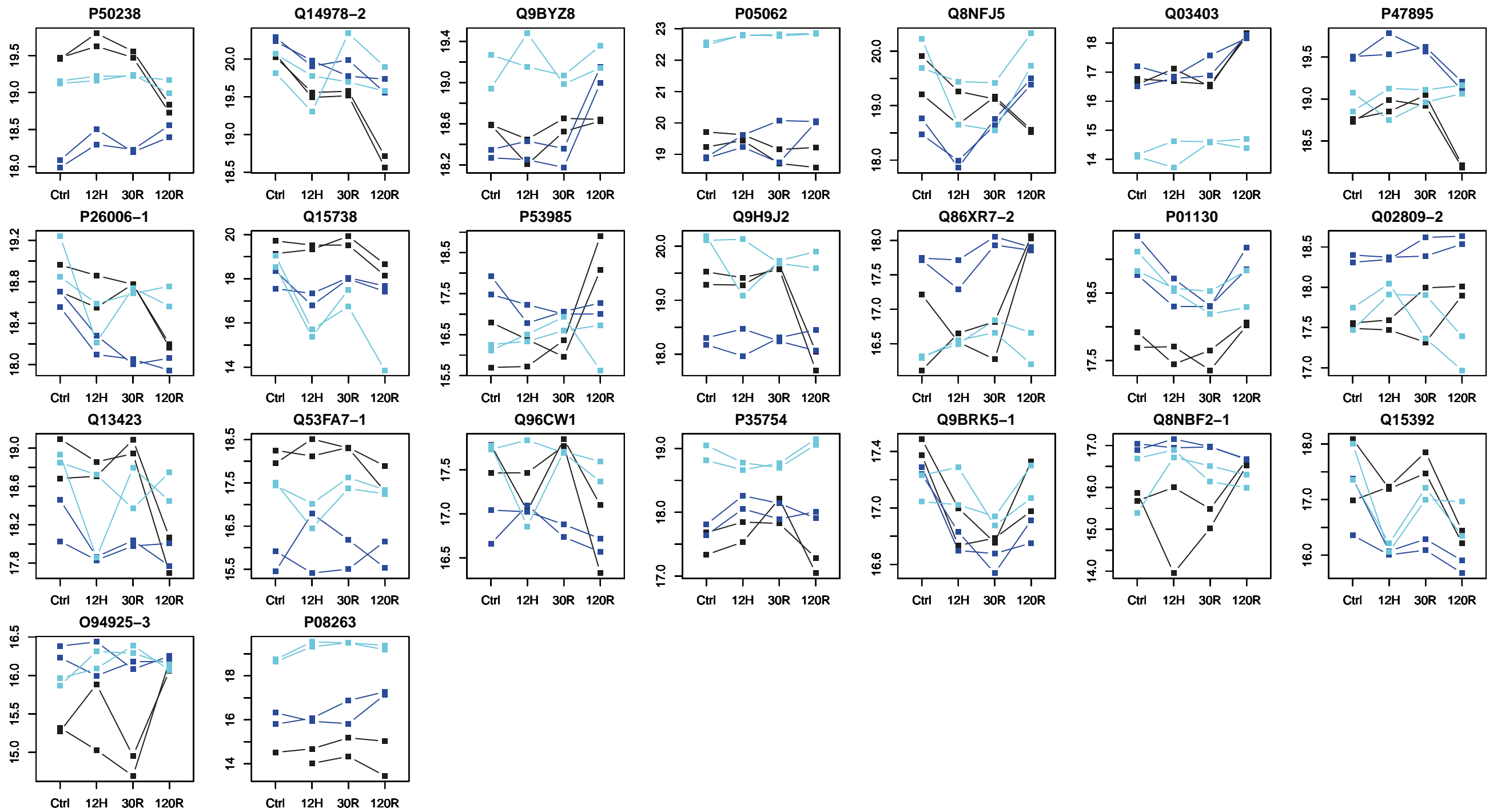

**C**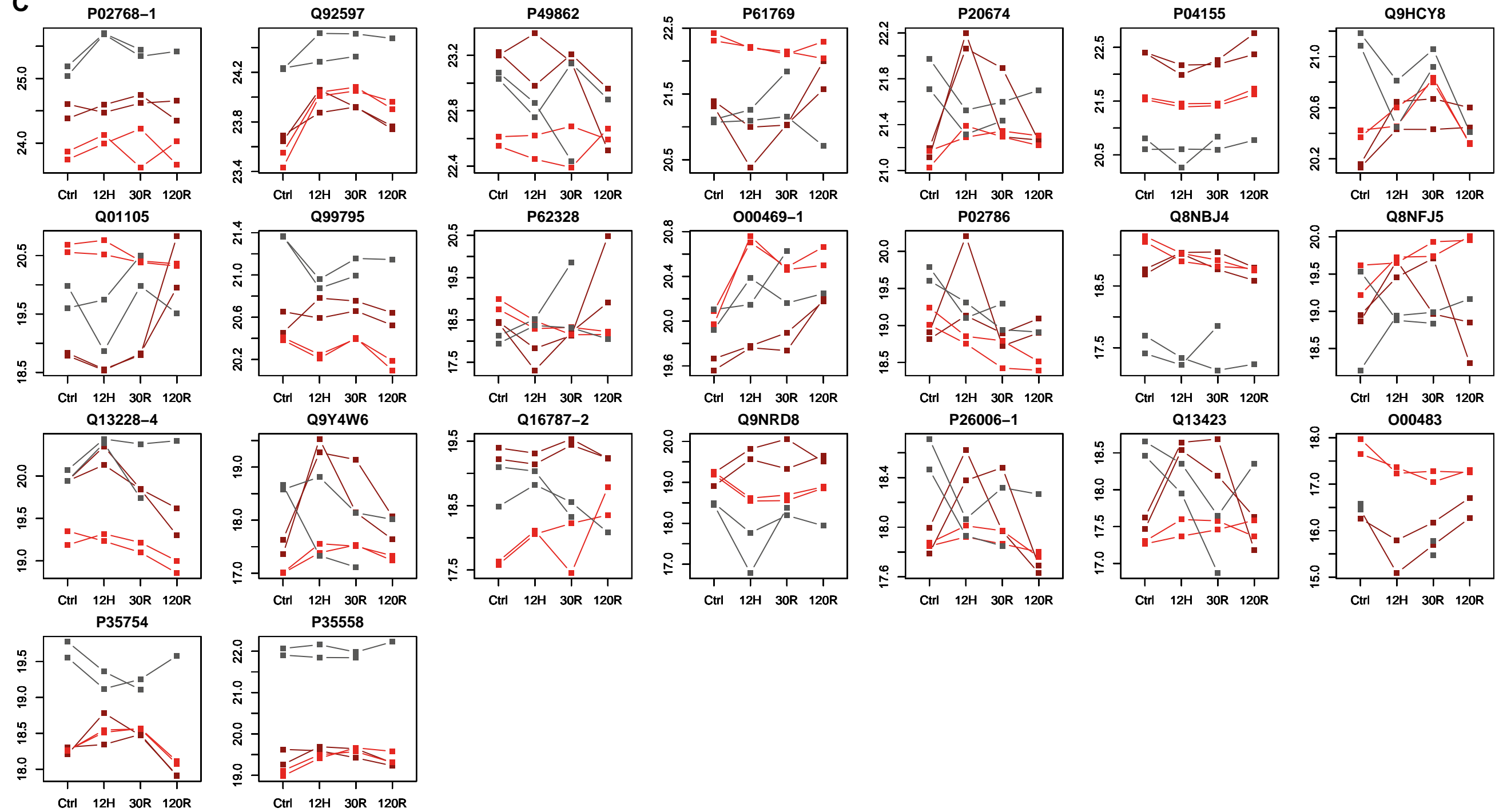

Supplement: Supplementary file 5 — Figure S4 [file 41419_2020_3379_MOESM5_ESM.pdf]
